# Psychometric Properties of the Arabic Version of the Pictorial Empathy Test for Assessing Affective Empathic Reactions in Patients with Schizophrenia

**DOI:** 10.3390/healthcare13162022

**Published:** 2025-08-16

**Authors:** Georges Kerbage, Camille Akkari, Nagham Hachem, Michelle El Murr, Rita El Mir, Cyril Abou Atme, Georges Haddad, Rony Abou Khalil, Elissar El Hayek, Frederic Harb, Souheil Hallit, Feten Fekih-Romdhane

**Affiliations:** 1 School of Medicine and Medical Sciences, Holy Spirit University of Kaslik, Jounieh P.O. Box 446, Lebanonsouheilhallit@usek.edu.lb (S.H.); 2 Faculty of Arts and Sciences, Holy Spirit University of Kaslik, Jounieh P.O. Box 446, Lebanon; 3 Psychiatry Department, Psychiatric Hospital of the Cross, Jal Eddib P.O. Box 60096, Lebanon; 4 Faculty of Medicine and Medical Sciences, University of Balamand, Kalhat P.O. Box 100, Lebanon; 5 Department of Psychology, College of Humanities, Effat University, Jeddah 21478, Saudi Arabia; 6 Applied Science Research Center, Applied Science Private University, Amman 11937, Jordan; 7 The Tunisian Center of Early Intervention in Psychosis, Department of Psychiatry “Ibn Omrane”, Razi Hospital, Manouba 2010, Tunisia; 8 Faculty of Medicine of Tunis, Tunis El Manar University, Tunis 1006, Tunisia

**Keywords:** schizophrenia, pictorial empathy test, validation, psychometric properties, Arabic

## Abstract

**Background/Objectives**: Although people with schizophrenia appear to experience emotions like healthy individuals, previous studies suggest that their ability to engage in empathic emotional responses might be impaired. As per our knowledge, no studies in the Arab world have investigated empathy in patients with schizophrenia, which is likely due to the lack of valid and reliable measures to assess the empathy construct among Arabic-speaking people. The aim of this research is to validate the Arabic version of the Pictorial Empathy Test (PET) in patients with schizophrenia from Lebanon. **Methods**: A two-month cross-sectional study was carried out at the Psychiatric Hospital of the Cross during January and February of 2024. The average age of the 113 participants in this study was 57.52 ± 10.35 years and 63.5% of them were men. Data were collected through in-person interviews. A confirmatory factor analysis (CFA) was conducted using SPSS AMOS version 29. Parameter estimation utilized the maximum likelihood approach. In order to examine sex invariance in PET scores, a multi-group CFA was conducted. Measurement invariance was assessed across configural, metric, and scalar levels. Evidence of invariance was determined based on the criteria: ΔCFI ≤ 0.010, ΔRMSEA ≤ 0.015, or ΔSRMR ≤ 0.010. **Results**: CFA revealed that the Arabic PET exhibited a unidimensional factor structure. The PET demonstrated solid internal consistency (ω = 0.93, α = 0.93). Measurement invariance testing confirmed that the scale performed equally well across sexes. A linear regression analysis found that female sex and higher levels of alexithymia were significantly correlated with lower levels of affective empathy. **Conclusions**: The findings indicate that the Arabic version of the PET is a reliable and valid tool for measuring affective empathy in Arabic-speaking patients with schizophrenia. The culturally adapted and validated Arabic PET would help detect affective empathy deficits, design and implement context-tailored interventions, and encourage future research in this area in the Arab region. Future research should aim to validate the PET against behavioral tasks like the Empathic Accuracy Task to improve its ecological validity.

## 1. Introduction

Empathy is a basic personal ability and a significant contributor to the formation and persistence of a healthy interpersonal relationship. Empathy refers to emotional reactions linked to others’ emotions and situations that are in accordance with others’ emotional states [1]. Traditionally, empathy encompasses two components reflecting both feeling and thinking in relation to others’ emotional states: affective empathy, which refers to experiencing others’ emotional states and cognitive empathy, which refers to understanding others’ emotions [2,3].

Empathy is decreased in the case of several diseases, such as traumatic brain injury, psychopathy, and schizophrenia [4,5,6]. Schizophrenia is a complex, chronic, severe mental health condition, affecting around one percent of the global population [7,8]. First described by Swiss psychiatrist Eugen Bleuler in 1908, schizophrenia is characterized by a variety of symptoms, including negative, positive, and cognitive ones [9,10]. Empathy is one social cognitive domain that is widely acknowledged to be compromised in schizophrenia [6]. It is a vague concept, defined as the capacity to comprehend and share the feelings, experiences, and emotions of others, as if they were one’s own [11,12]. Empathy can be further classified as affective (emotional), cognitive, and somatic [13]. We will only cover affective empathy in this study, which is defined as the capacity to share another person’s emotions [14].

Although people with schizophrenia appear to experience emotions like healthy individuals, previous studies suggest that their ability to engage in empathic emotional responses might be impaired [15]. A meta-analysis found a decrease in affective empathy in schizophrenia, although it was based on only six studies and did not provide a comprehensive review [16]. Subsequent research has confirmed this deficit, while other studies found no significant difference [16,17,18,19]. Notably, performance-based measures have shown stronger effects than self-report scales, indicating that the relationship between schizophrenia and affective empathy may vary depending on the type of measurement used [6]. In addition, affective empathy is often impaired in individuals with alexithymia, a condition that affects approximately 30% of people with schizophrenia [20,21]. It is characterized by trouble fantasizing and struggling in recognizing and describing emotions [22]. Patients with more severe alexithymia are primarily focused on external stimuli and are unaware of their own feelings [23]. A study found that deficits in processing speed, working memory, and abstract thinking were linked to difficulties in identifying feelings and external thinking [23].

Affective empathy is essential to social interactions because it encourages altruistic behavior and healthy relationships [24]. Reduced empathic ability in patients with schizophrenia could lead to unemployment and inability to lead an independent life [25]. Affective empathy is also thought to play a significant role in social functioning, as deficits in this domain may affect social interactions and contribute to the various social symptoms, including social withdrawal, often seen in individuals with schizophrenia [26,27]. In addition, it is reported that affective empathy promotes prosocial behavior, improved sleep quality, decreased rates of bullying, less violent behavior, and less substance abuse [28,29,30,31,32]. Given the influence of affective empathy on shaping social behaviors and relationships [28], it is important to accurately assess this construct, particularly in clinical populations such as individuals with schizophrenia, where impairments in empathy can lead to significant social and emotional difficulties [6,28,33]. Understanding these deficits in affective empathy is crucial for improving social functioning and developing effective interventions for this population. Many tools have been used to assess affective empathy, with controversial results across studies, depending on the tool being used [6]. This is mainly because empathy, as previously mentioned, is a vague and complex concept, and each tool may be designed to capture one aspect of it. Furthermore, it was shown that performance-based measures were associated with more authentic results as compared to self-report measures [6]. This might be due to the social desirability bias, which can occur in self-report (text-based) measures, where participants might answer in a way they perceive as socially acceptable rather than reflecting their true feelings [34].

These inconsistencies and challenges highlight the importance of carefully selecting appropriate tools for measuring affective empathy, particularly in clinical populations. In light of this, the following section will review specific instruments commonly employed in schizophrenia research to assess affective empathy.

### 1.1. Measurement of Affective Empathy in Patients with Schizophrenia

Behavioral measures represent a possible method for assessing affective empathy in patients with schizophrenia. These methods included tools like “Faux Pas Task” as well as “Reading the Mind in the Eyes Task”, which are static, meaning they measure empathy at one point in time. However, since empathy does not occur in a static environment, it is unclear if static tools can accurately represent a dynamic concept like empathy [35,36,37,38]. This led to the foundation of scales like the “Empathic Accuracy Task scale”, which captures the dynamic elements related to affective empathy [39]. This is achieved by screening different videos, where participants continuously assess scenes that are emotionally charged [38]. While being useful in assessing affective empathy, these tools are costly and time-consuming [40].

Furthermore, one of the most popular methods in evaluating empathy in patients with schizophrenia is self-report questionnaires, such as the “Interpersonal Reactivity Index” (IRI) and the “Questionnaire of Cognitive and Affective Empathy” (QCAE), which are commonly used and validated in several countries [41,42]. The IRI, known for its ability to evaluate the various aspects of empathy, is composed of four subscales; two for measuring emotional empathy and the other two are for measuring cognitive empathy [43]. Despite being widely used, the IRI was criticized for its outdated design and psychometric flaws, such as confusing empathy and sympathy [44]. The QCAE, which is relatively a newer instrument, was created from various already existing self-report scales, including the IRI, and was validated for use in people with schizophrenia [44,45]. This scale includes 5 subscales and is composed of 31 items, designed to assess, as its name implies, cognitive and affective empathy [45]. Given the need for culturally and cognitively accessible instruments, in addition to the tools mentioned above, the Pictorial Empathy Test (PET), a self-report tool, has emerged as a promising alternative for assessing affective empathy.

### 1.2. The Pictorial Empathy Test

The PET was first created and validated in the Finnish language by Lindeman [46]. This instrument can be used for the assessment of affective empathy. Participants rate the emotional impact of images of distressed and vulnerable people in different scenes. It is a scale composed of seven different pictures and validated in several languages, including Spanish and English, where they both showed similar psychometric features to the original measure by Lindeman [47,48]. Originally, when tested in several groups of adults, the PET showed a single factor structure, sufficient convergent validity, and good internal consistency [46]. Moreover, the PET has only very recently been translated and validated into Arabic among a random sample of young adults in Tunisia, where it also showed satisfactory psychometric properties, including strong internal consistency, good construct validity, and a clear factor structure [49]. In addition to being validated, this scale has been used in several studies globally, including Turkey and Taiwan [50,51].

Apart from its psychometric characteristics, the PET has several advantages over classic self-reported questionnaires. Since it consists of images, this visual format makes this test more universally accessible, reducing issues related to language barriers and cultural differences in interpretation. By relying on images rather than written statements and depending less on language, PET offers a more reliable method for assessing empathy across diverse populations. Furthermore, this brief test decreases the amount of space it takes up in surveys and lowers the possibility of participant tiredness, which could impact how a person might answer surveys [52]. Furthermore, participants may complete this test more quickly and easily since it is short, in contrast to long questions that can be time-consuming. Lastly, as this scale uses visual images instead of sentences, it is a more accurate way to measure affective empathy than simply asking participants how they feel when others are upset or distressed [46].

### 1.3. Rationale of This Study

As per our knowledge, no studies in the Arab world have investigated empathy in patients with schizophrenia, which is likely due to the lack of valid and reliable measures to assess the empathy construct among Arabic-speaking people. Indeed, most of the scales that assess empathy in patients with schizophrenia were originally developed and validated in European and American countries. These countries’ cultural contexts differ significantly from that of the Arab world. Individualists and collectivists may express emotions differently both within and between cultures [53]. People living in countries that tend to follow the individualist culture, like the United States or Australia, usually tend to express feelings more easily and openly, compared to countries that follow the collectivist. Middle Eastern and most Arabic-speaking populations tend to follow the collectivist culture, raising concerns about the validity of applying Western validated scales in our context [54]. Several healthcare systems in the Middle East have not yet reached their full potential because of economic crises, rapid population growth, and political issues, which could affect the diagnosis and management of several mental disorders, including schizophrenia [55,56]. In fact, in Lebanon, a lack of personnel was evident, as for one hundred thousand people, almost only one psychiatrist is available [57]. Because the PET is an economic and time-efficient tool for administrators and respondents, it is especially appropriate for settings with minimal resources, like many Arab contexts. Furthermore, patients with schizophrenia are often subject to language comprehension deficits [58]. Therefore, validating a scale that assesses empathy by relying only on images would be essential, as it minimizes the risk of misunderstanding because of cognitive or language barriers. Hence, we aimed at examining the psychometric properties of the Arabic version of the PET and assessing its correlates in patients with schizophrenia. Our first hypothesis is that this scale will demonstrate a single-factor structure. Additionally, we are expecting that this tool will demonstrate high reliability and strong construct validity.

## 2. Materials and Methods

### 2.1. Study Design

During a two-month period, lasting from January until February 2024, we conducted a cross-sectional study at the Psychiatric Hospital of the Cross, Jal El Dib, a hospital with more than nine hundred beds delivering care for psychiatric patients [59]. It is considered the biggest psychiatric hospital in the country and includes short- and long-stay services, as well as outpatient clinics [59]. A total of 133 Lebanese inpatients with schizophrenia were approached according to a list provided by the hospital administration. We included in our study Lebanese patients aged eighteen or older with a schizophrenia diagnosis based on the DSM-5, who had been hospitalized for more than one year and were therefore considered long-stay patients [59,60]. Patients with schizoaffective disorder and patients who declined to participate were not included. Out of the 133 patients approached, 52 females and 81 males were invited to participate in our study. A total of 11 females and 9 males declined to complete the questionnaire. Out of the twenty patients who were excluded, two patients were not able to complete the second section for isolation purposes, one patient could not complete the second section for work-related purposes, and seventeen were excluded as they did not give consent and therefore refused to complete either the first or second section of the questionnaire. Patients did not receive any compensation for their participation and were informed about the option of leaving the study at any time.

### 2.2. Data Collection

Data were gathered through face-to-face interviews and the Arabic questionnaire, which consisted of both closed and open-ended questions, was administered by five different students. Those students underwent a standardized training session covering ethical considerations, interview techniques, and uniform administration procedures of the survey questions. The questionnaire was divided into two big sections, each completed on a different day, to minimize fatigue, with each section taking approximately twenty minutes to complete. The total time for completing both sections was around thirty to forty minutes per patient. The first section included questions related to sociodemographic characteristics: age, gender, marriage status, education level, length of illness, and length of hospitalization. Additionally, patients answered the items of different scales. The other measures were selected based on both theoretical and empirical research suggesting their potential associations with empathy in schizophrenia, as mentioned above. They were also chosen for their strong psychometric properties and prior validation in Arabic-speaking populations. Each scale targets key constructs relevant to schizophrenia, including interviewer-assessed symptom severity and social functioning, as well as self-reported alexithymia.

#### 2.2.1. The PET Scale

As previously discussed, it is a visual scale where seven pictures of people in distress, males, females, and kids, are shown [46]. After each picture, patients were given a few seconds and were asked to rate how touching the imaging from a scale ranging from one to five, with one equivalent to “not at all”, and five, equivalent to “very much”. The scale consists of free-to-use images and was translated and validated recently for use in the Arabic-speaking population, where it showed solid psychometric features in a group of young Tunisian adults [49].

#### 2.2.2. The Positive and Negative Syndrome (PANSS) Scale

The PANSS is designed to assess the degree of symptom severity in patients with schizophrenia [61]. The questionnaire includes a total of thirty items and is composed of sixteen items for general psychopathology, seven items for positive and negative symptoms each. This scale has been validated in Arabic [62]. (Cronbach’s α in this study = 0.92)

#### 2.2.3. The Global Assessment of Functioning (GAF) Scale

The GAF is used in patients with schizophrenia for evaluating their social functioning levels [63]. Each patient is given a number between zero and one hundred by the interviewer, with higher scores corresponding to a better state. Because all the interviewers were trilingual, the scale was used in English.

#### 2.2.4. The Toronto Alexithymia Scale (TAS-20)

This scale was used to measure alexithymia. Each item is scored using the 5-point Likert scale, with scores ranging from one, which corresponds to “strongly disagree” to five corresponding to “strongly agree” [64]. TAS-20 has been validated in Arabic [65]. (Cronbach’s α in this study = 0.78)

### 2.3. Analytic Strategy

Confirmatory factor analysis. A confirmatory factor analysis (CFA) was conducted on the complete dataset, which had no missing values. Analysis was performed using SPSS AMOS version 29. Following established guidelines [66], the target sample size was determined to be between 21 and 140 participants, representing three to twenty times the number of scale variables. Parameter estimation utilized the maximum likelihood approach. Model fit assessment incorporated multiple indices: the Tucker–Lewis Index (TLI), standardized root mean square residual (SRMR), root mean square error of approximation (RMSEA), and comparative fit index (CFI). Acceptable model fit was defined by the following criteria [67]: RMSEA and SRMR values not exceeding 0.08 and 0.05, respectively, while CFI and TLI values needed to meet or exceed 0.90. Due to the initial absence of multivariate normality (Bollen–Stine bootstrap *p* = 0.002), the analysis implemented non-parametric bootstrapping procedures.

Sex invariance. Using the full sample, and in order to examine sex invariance in PET scores, a multi-group CFA was conducted [68]. Measurement invariance was assessed across configural, metric, and scalar levels [69]. Evidence of invariance was determined based on the criteria: ΔCFI ≤ 0.010, ΔRMSEA ≤ 0.015, or ΔSRMR ≤ 0.010 [70]. The student *t*-test was employed to compare two means.

Composite reliability was evaluated using Cronbach’s α as well as McDonald’s ω, with values exceeding 0.70 indicating sufficient reliability. Normality was confirmed as skewness and kurtosis values for each scale item ranged between −1 and +1 [71]. The Pearson correlation test was applied to examine the relationship between PET scores and other survey scales. To identify factors associated with empathy, a linear regression analysis was performed. Variables with a *p*-value below 0.25 in the bivariate analysis were included as independent variables in the final model. This more inclusive threshold was chosen to avoid excluding potentially important predictors too early in the modeling process, as recommended in model-building strategies [72]. Statistical significance was defined as *p* < 0.05.

## 3. Results

### 3.1. Participants

Among the 113 patients who took part in the study, their average age was 57.52 ± 10.35 years and 63.5% of them were male. Table 1 summarizes the other characteristics.

### 3.2. Confirmatory Factor Analysis

**Figure 1:**
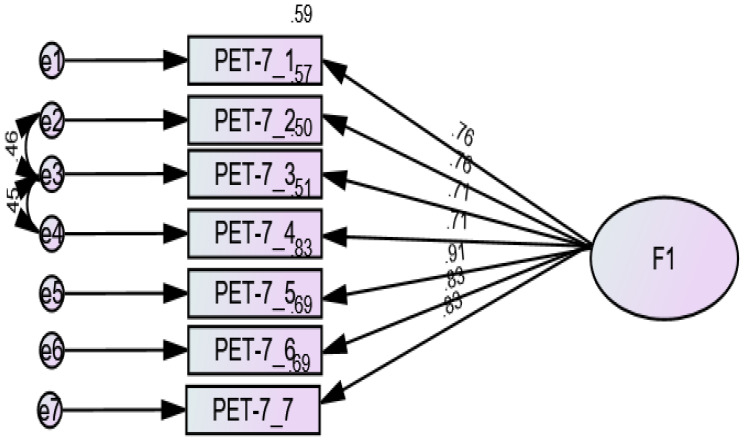
Standardized factor loadings of the Arabic version of the 7-item Pictorial Empathy Test.

CFA was conducted to evaluate the PET scale’s one-factor structure. Initial analysis revealed moderate model fit statistics (TLI = 0.832; CFI = 0.888; SRMR = 0.054; RMSEA = 0.204, with 90% confidence interval ranging from 0.162 to 0.249). Further examination identified high modification indices (>10), suggesting correlations between items 2–3 and 3–4 (Figure 1). Upon incorporating these correlations, the model showed substantial improvement in fit metrics (TLI = 0.943; CFI = 0.967; SRMR = 0.041; RMSEA = 0.119, 90% CI: 0.068–0.171). Both omega and alpha coefficients (0.93) indicated strong internal consistency.

### 3.3. Sex Invariance

The results, as shown in Table 2, indicate that sex invariance was achieved at the configural, metric, and scalar levels. Females demonstrated significantly lower mean empathy scores compared to males (18.29 ± 6.86 vs. 24.14 ± 7.37; t(111) = 4.16; *p* < 0.001; Cohen’s d = 0.813).

### 3.4. Bivariate Analysis

The relationships between empathy and several factors were examined through bivariate analyses, with results detailed in Table 3 and Table 4. The level of education was not significantly associated with empathy (Table 3), whereas higher alexithymia was significantly associated with lower empathy (r = −0.46; *p* < 0.001).

### 3.5. Multivariable Analysis

The linear regression analysis, with empathy score as the dependent variable, identified female sex (Beta = −3.41) and elevated alexithymia levels (Beta = −0.21) as significant predictors of lower empathy (Table 5).

## 4. Discussion

The Arabic version of the PET demonstrated solid psychometric properties, including good reliability and validity, making it a great instrument for assessing empathy in Arabic-speaking patients with schizophrenia. Confirmatory factor analysis validated the single-factor structure of the instrument, achieving satisfactory fit indices after minimal item-specific adjustments. Also, the Arabic version of the PET showed a strong concurrent validity, as it was significantly and negatively correlated with alexithymia.

### 4.1. Factor Structure and Internal Consistency of the PET

First, a unidimensional factor structure was revealed by the results of the CFA for the Arabic version of the PET, which aligns with the original version of this scale, as well as previous validations of the PET in other languages [46,47,49]. The fact that the factor loadings were higher than the minimum suggested cutoff of 0.30 indicates that each of the 7 items contributes significantly to the overall empathy construct [73]. Although some model fit indices showed moderate initial values, the model improved after adjusting for correlated item pairs (items 2–3 and 3–4), with the fit indices reaching excellent values. This comes in favor of this scale is a valid tool for the examination of empathy in Arabic-speaking populations, including patients with schizophrenia, in whom measuring empathy can be challenging due to cognitive and affective abnormalities [6].

In addition, the PET’s Arabic version has shown excellent internal consistency, with composite reliability coefficients (ω = 0.93 and α = 0.93), which are well above the commonly accepted threshold of 0.70 for acceptable reliability, suggesting that the scale provides consistent results [74].

Furthermore, the RMSEA value remained above the common threshold of 0.08 even after adding the correlations. It is important to note that RMSEA tends to be inflated in models with low degrees of freedom (df < 50) [75,76]. Our model has a df of 12, implying that RMSEA may not serve as a reliable fit index in this context. Therefore, greater emphasis should be placed on alternative fit indices such as CFI and SRMR, which are considered more stable under these conditions [77]. In our study, both CFI and SRMR values indicated an adequate model fit, thereby supporting the overall suitability of the model despite the increased RMSEA.

### 4.2. Measurement Invariance Across Sex

According to measurement invariance testing, the Arabic version of the PET performed equally in both males and females, with consistent factor structure and model fit across sexes. This result indicates that PET is a suitable instrument for use in both sexes. Findings in our study revealed that males and females had significantly different empathy scores, with females showing lower empathy than males. Lower levels of affective empathy in female patients with schizophrenia may contribute, as previously mentioned, to greater social dysfunction and difficulties in maintaining relationships, which are already prominent challenges in this population [28]. Previous studies have shown that several factors, including sex, culture, and social influences, might affect empathy [74]. In fact, while it is a common belief that females are more empathetic than males, research findings were controversial regarding this statement [75]. For example, few studies found that there is no difference between sexes, while others have found that females have greater empathy levels, especially in studies where researchers assessed empathy using a self-report questionnaire [76,77,78].

Furthermore, in Arab societies, masculinity is shaped by cultural expectations that promote emotional control, strength, and resilience [78]. It was already shown that cultural orientations may influence how people perceive and exhibit empathy in various social circumstances. Individual behaviors and social interactions are frequently influenced by cultural norms, which people accept and internalize. Empathy is impacted by culture and related values, especially individualistic/collectivist norms in nations [79]. Men are often expected to suppress emotions and avoid showing vulnerability. This emotional suppression, while socially rewarded, can increase the risk for mental health problems over time [80]. Despite these pressures, men in our sample may have reported higher empathy because they were influenced by social expectations during the assessment. Therefore, their higher scores might not reflect how they truly feel.

In addition, while examining the raw data, we found that the average age of male participants with schizophrenia was 58.2 years, while that of female participants was 56.5 years. Age may also play a role in empathy levels. Some studies have shown that older individuals tend to report higher empathy, possibly due to the accumulation of life experiences over time [81]. Although the age difference between sexes in our sample is modest, it may have contributed, in part, to the observed variation in empathy scores and warrants further investigation in future research. In this regard, studies adopting qualitative interviews could explore how cultural norms could differently influence empathy expression across sexes. We acknowledge the unequal and relatively small subgroup sizes (72 males and 41 females) in our sample. While these findings suggest that the factor structure and item functioning are consistent across sexes, the limited sample size warrants caution in interpreting these results.

### 4.3. Concurrent Validity

The concurrent validity of the Arabic PET was examined by its correlation with alexithymia. Similar to what previous articles have shown, our study found that affective empathy and alexithymia were negatively correlated, meaning that whenever empathy is high, alexithymia is low and vice versa [82]. Individuals diagnosed with schizophrenia are known to struggle in connecting with others, as symptoms such as alexithymia impair social functioning [83,84]. Our findings indicate that those who struggle to recognize and understand their own feelings might have an altered capacity for empathy. In fact, alexithymia can make identifying and reacting to other people’s emotions a very difficult task [85]. It has been shown through neuroimaging studies that individuals who have alexithymia present lower activity levels in the brain that are related to empathy and feelings expression, which might explain why people with high alexithymia have low levels of empathy [86,87].

Furthermore, while previous studies have linked empathy with the severity of clinical symptoms in patients with schizophrenia, our findings did not show a significant correlation. A previous study revealed a negative correlation between PANSS scores and affective empathy, indicating that as symptom severity increases in schizophrenia, empathic abilities of a patient with schizophrenia decline [88]. This is consistent with other studies as well that have shown a link between empathy and certain symptoms of schizophrenia, specifically negative symptoms, which were related to overall empathy [26]. Other symptoms, like disorganized or depressive symptoms, have also been linked to empathy [17]. Researchers have also pointed to a substantial negative association between PANSS severity scores and empathy [89], but a lack of connection [26,90] or no more than a weak correlation [88] between positive symptoms and empathy have been reported as well. These mixed findings regarding the effect of psychotic symptoms on empathy are likely due to the high clinical heterogeneity of schizophrenia.

### 4.4. Study Limitations

First, participants in our study were exclusively Lebanese, which limits the ability to generalize the findings to other Arab regions with diverse cultural backgrounds and nationalities. Additionally, we used a cross-sectional design, data were gathered at a single point in time, which limited examination of the test-retest reliability and predictive validity. Our sample had a disproportionate gender representation, which might have distorted the results. Furthermore, antipsychotic medications are known to affect emotional response [91]. In our study, we did not account for the chlorpromazine dose equivalent of antipsychotics for each patient, which could therefore be considered a limitation, as even though it is beneficial for schizophrenia patients initially, higher doses of antipsychotics might lead to an increase in emotional blunting, affecting levels of affective empathy [92,93]. Lastly, we recruited chronic patients from only one hospital, which might lead to a selection bias. Future studies should recruit participants from multiple Arab regions and include both inpatient and outpatient populations to improve the generalizability of findings. Additionally, since the participants were long-stay patients, their limited social interactions because of prolonged hospitalization might restrict the variety of empathic situations they encounter, potentially affecting their empathy scores.

### 4.5. Study’s Relevance and Practical Implications

Notwithstanding these limitations, our study presented valuable clinical implications. First, we showed that the Arabic version of the PET scale is a valid and consistent scale for assessing empathy in Lebanese patients with schizophrenia. Clinically, using a culturally adapted measure enables more accurate detection of empathy deficits, which could inform the development of targeted psychosocial interventions aimed at enhancing affective empathy. Improving empathy in this population may help reduce social isolation and interpersonal difficulties.

In addition, given that affective empathy impairments are also central in other conditions such as autism spectrum disorder, the validated Arabic version of the PET may have potential utility for assessing empathy deficits in these populations as well. Future research could explore its applicability and validity in such clinical groups.

Additionally, our research highlights the importance of culturally tailored tools, which could help bridge the gap in mental health assessments across diverse populations. Improving affective empathy is a key for promoting better social interactions, which could reduce social isolation, both being critical factors for patients’ overall quality of life and subsequently a strong predictor of therapeutic success [89]. Lastly, validating for the first time a scale that assesses affective empathy in patients with schizophrenia would encourage future research in Lebanon and in Arabic-speaking populations on this important topic, which would fill the gap in the literature. Future research should focus on longitudinal studies to examine how affective empathy evolves over the course of schizophrenia, which would help clarify causal relationships and the potential impact of treatment.

## 5. Conclusions

By validating this tool, we provided physicians and researchers with a cost-effective, easy-to-use and reliable instrument that would help in assessing affective empathy in Arabic-speaking patients with schizophrenia. Moreover, our results aligned with those of the literature, indicating that affective empathy and alexithymia were inversely correlated. Further investigation is required to determine the causes behind the sex variations in affective empathy scores, particularly in cultural contexts in which sex stereotypes may modulate emotional behaviors. Lastly, given that the PET is a static, image-based tool, it may not fully capture the complexity of empathy in dynamic, real-life interactions. Therefore, future research should aim to validate the PET against behavioral tasks like the Empathic Accuracy Task to improve its ecological validity and take into consideration the antipsychotic medications.

## Figures and Tables

**Table 1:** Sample sociodemographic profile and characteristics (n = 113).

Variables	N (%)
Sex	
Males	72 (63.7%)
Females	41 (36.3%)
Level of Education	
Elementary	27 (23.9%)
Complementary	42 (37.2%)
High school	30 (26.5%)
University education	14 (12.4%)
	Mean ± SD
Age (years)	57.52 ± 10.35 [min = 33; max = 76]
Age of schizophrenia onset	25.33 ± 7.71 [min = 13; max = 48]
Length of hospitalization (years)	3.69 ± 4.82 [min = 0.01; max = 32]
Length of illness (years)	32.04 ± 11.59 [min = 6; max = 61]
Affective empathy	19.98 ± 7.70 [min = 7; max = 34]
PANSS	90.27 ± 28.08 [min = 30; max = 156]
GAF	71.59 ± 25.40 [min = 20; max = 100]
Alexithymia	53.58 ± 13.82 [min = 30; max = 90]

**Table 2:** Sex-based measurement invariance of the overall sample’s PET results.

Model	CFI	RMSEA	SRMR	Model Comparison	ΔCFI	ΔRMSEA	ΔSRMR
Configural	0.966	0.082	0.044				
Metric	0.967	0.073	0.043	Configural vs. metric	0.001	0.009	0.001
Scalar	0.977	0.056	0.046	Metric vs. scalar	0.010	0.017	0.003

Note. CFI refers to the Comparative Fit Index, RMSEA represents the Root Mean Square Error of Approximation, and SRMR stands for the Standardized Root Mean Square Residual.

**Table 3:** Bivariate analysis of empathy across different education levels.

	Mean ± SD	F	df1, df2	*p*	Effect Size
Level of Education		0.13	3, 109	0.944	0.003
Elementary	21.33 ± 9.16				
Complementary	22.07 ± 8.03				
Secondary school	22.60 ± 7.01				
University education	21.93 ± 5.27				

**Table 4:** Pearson correlation matrix of empathy and related variables.

	1	2	3	4	5	6	7
1. Affective empathy	1						
2. Age	0.09	1					
3. Age of schizophrenia onset	−0.17	0.17	1				
4. Duration of Hospital Stay	0.06	0.07	−0.17	1			
5. Duration of the disease	0.17	0.73 ***	−0.46 ***	0.04	1		
6. PANSS	0.10	0.05	0.05	0.04	−0.004	1	
7. GAF	−0.12	−0.13	−0.03	0.05	−0.08	−0.68 ***	1
8. Alexithymia	−0.46 ***	−0.01	0.08	−0.14	−0.04	0.09	−0.03

Note. Values marked with *** denote *p* < 0.001. Abbreviations used: Overall Functioning Assessment (GAF), Scale for Assessment of Positive and Negative Symptoms (PANSS).

**Table 5:** Linear regression analysis of factors associated with empathy in patients with schizophrenia.

	Unstandardized Beta	Standardized Beta	*p*	95% CI
Sex (females vs. males *)	−3.41	−0.21	**0.026**	−6.40; −0.42
Age of schizophrenia onset	−0.07	−0.07	0.471	−0.25; 0.12
Length of illness	0.04	0.06	0.553	0.09; 0.16
General functioning	−0.05	−0.17	0.051	−0.10; 0.001
Alexithymia	−0.21	−0.37	**<0.001**	−0.30; −0.11

* denotes reference group. Significant *p*-values are indicated by numbers in bold. Males were coded 0 and females were coded 1.

## Data Availability

The data that support the findings of this study are available from the corresponding author but restrictions apply to the availability of these data, which were used under license for the current study, and so are not publicly available. Data are however available from the authors upon reasonable request and with permission of the ethics committee.

## Abbreviations

The following abbreviations are used in this manuscript:

PET	Pictorial Empathy Test
IRI	Interpersonal Reactivity Index
QCAE	Questionnaire of Cognitive and Affective Empathy
TLI	Tucker-Lewis Index
SRMR	Standardized root mean square residual
RMSEA	Root mean square error of approximation
CFI	comparative fit index
PANSS	Positive and Negative Syndrome Scale
GAF	Global Assessment of Functioning scale
TAS-20	Toronto Alexithymia Scale
